# Temperature, phosphorus and species composition will all influence phytoplankton production and content of polyunsaturated fatty acids

**DOI:** 10.1093/plankt/fbad026

**Published:** 2023-06-24

**Authors:** Marco L Calderini, Salli Pääkkönen, Pauliina Salmi, Elina Peltomaa, Sami J Taipale

**Affiliations:** Department of Biological and Environmental Science, University of Jyväskylä, P.O. Box 35 FI-40014, Jyväskylä, Finland; Spectral Imaging Laboratory, Faculty of Information Technology, University of Jyväskylä, P.O. BOX 35 FI-40014, Jyväskylä Finland; Spectral Imaging Laboratory, Faculty of Information Technology, University of Jyväskylä, P.O. BOX 35 FI-40014, Jyväskylä Finland; Department of Forest Sciences, University of Helsinki, P.O. Box 27 FI-00014, Helsinki, Finland; Department of Biological and Environmental Science, University of Jyväskylä, P.O. BOX 35 FI-40014, Jyväskylä, Finland

**Keywords:** phytoplankton, polyunsaturated fatty acids, lake, climate change, temperature, phosphorus

## Abstract

Temperature increases driven by climate change are expected to decrease the availability of polyunsaturated fatty acids in lakes worldwide. Nevertheless, a comprehensive understanding of the joint effects of lake trophic status, nutrient dynamics and warming on the availability of these biomolecules is lacking. Here, we conducted a laboratory experiment to study how warming (18–23°C) interacts with phosphorus (0.65–2.58 μM) to affect phytoplankton growth and their production of polyunsaturated fatty acids. We included 10 species belonging to the groups diatoms, golden algae, cyanobacteria, green algae, cryptophytes and dinoflagellates. Our results show that both temperature and phosphorus will boost phytoplankton growth, especially stimulating certain cyanobacteria species (*Microcystis* sp.). Temperature and phosphorus had opposing effects on polyunsaturated fatty acid proportion, but responses are largely dependent on species. Eicosapentaenoic acid (EPA) and docosahexaenoic acid (DHA) synthesizing species did not clearly support the idea that warming decreases the production or content of these essential polyunsaturated fatty acids. Our results suggest that warming may have different effects on the polyunsaturated fatty acid availability in lakes with different nutrient levels, and that different species within the same phytoplankton group can have contrasting responses to warming. Therefore, we conclude that future production of EPA and DHA is mainly determined by species composition.

## INTRODUCTION

Lakes respond quickly to environmental change by altering their physical, chemical and biological properties, making estimations about the fate of these ecosystems complex (Adrian *et al.*, 2009). Temperatures across the globe are expected to increase due to climate change (IPCC, 2021) leading, in theory, to more productive aquatic ecosystems (Falkowski and Raven, 2007). Nevertheless, lake nutrient status and dynamics, morphological characteristics and light availability are likely to play a role in modulating the effects of temperature increases in these ecosystems (Adrian *et al.*, 2009; Jennings *et al.*, 2009; Björnerås *et al.*, 2017; Tabari, 2020). In high-latitude regions of the northern hemisphere, lakes are observed in high frequencies and provide significant ecosystem services in addition to habitats for wildlife (Chapin *et al.*, 2004). In northern areas, climate change is expected to increase precipitations, facilitating the run-off of nutrients (phosphorus and nitrogen) and dissolved organic carbon from catchment areas. This can result in eutrophication and browning of surface waters (de Wit *et al.*, 2016, Ruosteenoja *et al.*, 2016). Therefore, understanding how temperature increases and nutrients interact to shape lake responses is key when making estimations about the fate of northern lakes.

Phytoplankton provides aquatic food webs with energy, high-quality biochemical compounds and minerals (Sterner and Hessen, 1994; Peltomaa *et al.*, 2017). Temperature and nutrient increases have been shown to alter phytoplankton total biomass, community structure and the biochemical composition of individual cells (Adrian *et al.*, 2006; Rosenzweig *et al.*, 2007; Winder and Hunter, 2008; Taipale *et al.*, 2019). This is especially important when considering the availability of certain micronutrients present in phytoplankton that are essential for higher trophic levels. For example, long-chain polyunsaturated fatty acids (LC-PUFAs) such as eicosapentaenoic acid (EPA, 20:5ω-3) and docosahexaenoic acid (DHA, 22:6ω-3) are only produced by certain phytoplankton taxa but are required for the appropriate development and reproduction of consumers (Arts *et al.*, 2001; Parrish, 2009). Consequently, phytoplankton community alterations and changes in cellular contents reducing LC-PUFA availability can have cascading effects for higher trophic levels (Ahlgren *et al.*, 1992; Lang *et al.*, 2011; Taipale *et al.*, 2013). Currently, it is hypothesized that water warming will decrease PUFA availability, in particular EPA and DHA (Hixson and Arts, 2016; Colombo *et al.*, 2019), due to the overall decrease in fatty acid unsaturation degree observed across temperature gradients (Sepúlveda and Cantarero, 2022). These observations are conceptually validated by the homeoviscous adaptation theory (Sinensky, 1974), which states that at high temperatures saturated fatty acids give stability to cellular membranes. Over large temperature gradients, we believe that this theory holds since membranes originally adapted to cold environments are unstable at high temperatures. Nevertheless, nutrients such as phosphorus and nitrogen can also strongly modulate PUFAs (Su *et al.*, 2016; Ghafari *et al.*, 2016; Wang *et al.*, 2019), EPA and DHA (Xu *et al.*, 2001; Khozin-Goldberg and Cohen, 2006; Ren *et al.*, 2012; Matsui *et al.*, 2020) due to their participation in cellular metabolism and synthesis of certain lipid classes (Van Mooy *et al.*, 2009). Therefore, within the temperature increase expected with climate change, nutrients could play a significant role in the availability of PUFAs, leading to divergent scenarios than previously proposed (Hixson and Arts, 2016; Colombo *et al.*, 2019).

In lakes, phosphorus is a key macronutrient strongly associated with phytoplankton growth (Schindler, 1977). Given that phosphorus is a building block of membrane lipids (Van Mooy *et al.*, 2009; Cañavate *et al.*, 2016), and cell growth is interconnected with lipid metabolism (Tsai *et al.*, 2014), phosphorus concentration modulates phytoplankton PUFA, EPA and DHA availability (Khozin-Goldberg and Cohen, 2006; Ren *et al.*, 2012; Matsui *et al.*, 2020). No general effect of increasing phosphorus on phytoplankton LC-PUFAs has been observed, pointing to the diversity of phytoplankton life histories (Lubchenco and Cubit, 1980; Cock *et al.*, 2014) and highlighting that different species can present contrasting responses to changes in this nutrient (Adrian *et al.*, 2006). To date, most studies centred in the effect of phosphorus on phytoplankton LC-PUFAs have focused on nutrient depletion due to its applications in biotechnological processes (Khozin-Goldberg and Cohen, 2006; Ghafari *et al.*, 2016; Matsui *et al.*, 2020; Rawat *et al.*, 2021). Unfortunately, such an experimental approach completely overlooks phytoplankton responses to variations in phosphorus under non-depleted conditions, which could uncover valuable information about how differences in trophic status could affect LC-PUFAs availability in lakes.

We tested how simultaneous increases in temperature and phosphorus affect the growth, PUFAs and the LC-PUFAs (EPA and DHA) of 10 phytoplankton species common to northern lakes from six different phytoplankton groups. For this purpose, we use low (18°C) and high (23°C) growing temperatures combined with low (LP) and high (HP) available phosphorus to measure how phytoplankton growth rate, PUFA proportion, EPA and DHA content and production (measured as daily gain) are affected by these physicochemical changes. The experimental design was fully factorial. The objective of this study was to investigate the interaction between temperature increase and phosphorus in PUFA and LC-PUFA availability. Our phosphorus treatments served as a proxy to study the effect of increasing temperature at different trophic status, as well as the effects of increasing phosphorus at different temperatures. We hypothesize that the effect of increasing temperature on phytoplankton LC-PUFA is dependent on phosphorus concentration (Khozin-Goldberg and Cohen, 2006; Ren *et al.*, 2012; Matsui *et al.*, 2020) and that there are large differences in responses between phytoplankton species due to their different life histories (Lubchenco and Cubit, 1980; Adrian *et al.*, 2006; Cock *et al.*, 2014).

## MATERIALS AND METHODS

### Strains, culture preparation and growing conditions

Ten species from the phytoplankton groups diatom (*Cyclotella* sp. and *Melosira* sp.), chrysophyte (*Synura* sp. and *Uroglena* sp.), cyanobacteria (*Microcystis* sp. and *Synechococcus* sp.), green algae (*Chlamydomonas reinhardtii* and *Desmodesmus maximus*), cryptophyte (*Rhodomonas* sp.) and dinoflagellate (*Peridinium cinctum*) were acclimatized to low phosphorus and 18°C before the start of the experiment. For such purposes, phytoplankton species were maintained autotrophically in the authors’ culture collection as stock cultures in phosphorus-limited MWC (Modified Wright’s Cryptophyte) media (Guillard and Lorenzen, 1972), at a phosphorus concentration of 6.46 μM, at 18°C under a 12:12-h light–dark cycle (light intensity of 100–125 μmol quanta m^−2^ s^−1^). The experiment was divided into two halves to ensure proper experimental handling given the large number of phytoplankton cultures (120 cultures in total). In the first half cyanobacteria, green algae and cryptophytes were grown in 250 mL plastic culture flasks containing a final volume of 175 mL composed of 75 mL of phytoplankton stock and 100 mL of MWC. Experimental phosphorus-modified WC was prepared according to the treatment as low phosphorus (LP: 0.65 μM) and high P (HP: 2.58 μM). Phosphorus was added weekly to maintain cultures at their respective concentrations (assuming that phosphorus was zero at the moment of addition) to simulate consistent concentrations and avoid the effect of phosphorus depletion. Phytoplankton were grown in FH-130 (Taiwan Hipoint) growth chambers set a 18° and 23°C with a 12:12-h light–dark cycle and a light intensity 91–132 μmol quanta m^−2^ s^−1^. In the second half of the experiment, diatoms, golden algae and dinoflagellates were grown in 600 mL plastic culture flasks containing a final volume of 400 mL composed of 100 mL of phytoplankton stock and 300 mL of the same experimental MWC as before. Growth chamber conditions, as well as phosphorus additions, were the same as for the first half of the experiment. In this half of the experiment, larger flasks were used to ensure enough biomass due to the lower biomass obtained from the stock cultures. The experimental design was fully factorial and each phytoplankton species in each treatment was prepared in triplicates in both halves of the experiment. Cell concentration was measured every 2–3 days using a flow cytometer (Guava easyCyte HT; Luminex). Experiment was terminated individually for each species once they reached stationary growth phase. Growth rate (day^−1^) was calculated from the change in cell density during the exponential growth phase according to the formula: growth rate = ln(N_2_/N_1_)/(t_2_ − t_1_), where N_2_ is the maximum measured cell density, N_1_ is the cell density at Day 0 of the experiment and (t_2_ − t_1_) is the time between the start of the experiment and the day where N_2_ was measured.

### Fatty acid analysis

Once all cultures of the same phytoplankton species reached stationary phase, phytoplankton cells were harvested by filtration through 3.0 μm cellulose nitrate membranes (Whatman, GE Healthcare), obtaining between 0.3 and 7.5 mg dry weight, depending on the species. Total lipids were extracted, and FA identified and analyzed as previously described (Calderini *et al.*, 2022) without dividing the sample into different fractions. Shortly, total lipids were extracted with chloroform/methanol/water (4:2:1) using sonication (10 min). After evaporation of solvents under a nitrogen stream, 1 mL toluene was added, and fatty acids were transesterified overnight (50°C) using methanolic H_2_SO_4_ (1%, v/v). FA methyl esters were analyzed with a gas chromatograph equipped with a mass detector (GC–MS; Shimadzu Ultra) using a DB-23 column (30 m × 0.25 mm × 0.25 μm; Agilent). Quantification of FAs was based on peak integration using gcsolution software (version 2.41.00, Shimadzu). Peak areas of FAs were corrected by using two internal standards (phospholipid FA C19:0 and free FA C23:0; Larodan) added before lipid extraction.

### Data analysis

The value of PUFA proportion was obtained by dividing the content of all fatty acids containing two or more unsaturations by the sum of mono- and saturated fatty acids. Univariate analysis of variance (dependent variable: growth rate, PUFA proportion, EPA and DHA content and daily gain) was done with ANOVA, and equality of variance was checked with Bartlett’s test. Pairwise comparisons were carried out with Tukey’s honestly significant difference test. If data presented a significant Bartlett’s test (unequal variances), Kruskal–Wallis rank sum test was performed to assess the effects of the studied treatments. Non-parametric pairwise comparisons were also carried out with Kruskal–Wallis test using Bonferroni correction. Permutational multivariate analysis of variance (PERMANOVA) based on the Bray–Curtis distance matrix and multivariate homogeneity of group dispersion (Anderson, 2006) were performed on multivariate fatty acid composition (proportion of each FA) data using treatment, group or species as factors. Due to the collapse of *Synura* sp. LP cultures (18° and 23°C) before the end of the experiment, these treatments were taken out of the analysis and only *Synura* sp. HP (18° and 23°C) cultures were analyzed with ANOVA. ANOVA and PERMANOVA analyses were carried out to determine the overall effect of temperature and phosphorus on all studied species, excluding the data obtained for *Synura* sp cultivated in LP treatments. Non-metric multidimensional scaling (nMDS) was employed to visualize multivariate FA patterns in response to changes in temperature and phosphorus across the studied phytoplankton species. The limit of statistical significance in all tests was set to α = 0.05. All statistical analyses were conducted using r (RStudio version 4.0.5) with either R base or vegan package (Oksanen *et al.*, 2018). Given the limitation of *P*-values as indicators of effect size (Wasserstein *et al.*, 2019), we used Glass′ Δ (Glass *et al.*, 1981) as a fair estimate of effect size of treatments (Lin and Aloe, 2020). This estimate standardizes the difference in mean values between a control and a test group with the standard deviation observed in the control. We present such values in this study as heatmaps, where for each comparison between two treatments, the first denoted treatment is considered as control to calculate Glass’ Δ.

## RESULTS

### Effect of temperature and phosphorus on growth rate

**Fig. 1:**
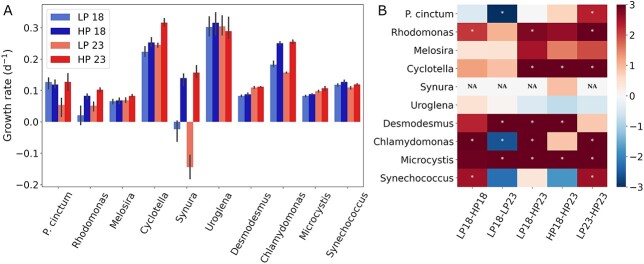
Growth rate (day^−1^) per species (**A**) and normalized growth rates (Glass′ Δ) changes between treatments using the first referred treatment as baseline (**B**). Phytoplankton species are representatives of the groups diatoms (*Cyclotella* sp. and *Melosira* sp.), golden algae (*Synura* sp. and *Uroglena* sp.), cyanobacteria (*Microcystis* sp. and *Synechococcus* sp.), green algae (*Chlamydomonas reinhardtii* and *Desmodesmus maximus*), cryptophytes (*Rhodomonas* sp.) and dinoflagellates (*Peridinium cinctum*). Treatment names correspond to culture condition with 18 and 23 denoting temperature in °C and LP and HP denoting phosphorus concentration [0.65 (LP) and 2.58 (HP) μM phosphorus]. * (white marker) denotes statistical difference between treatment comparison in each phytoplankton species.

**Fig. 2:**
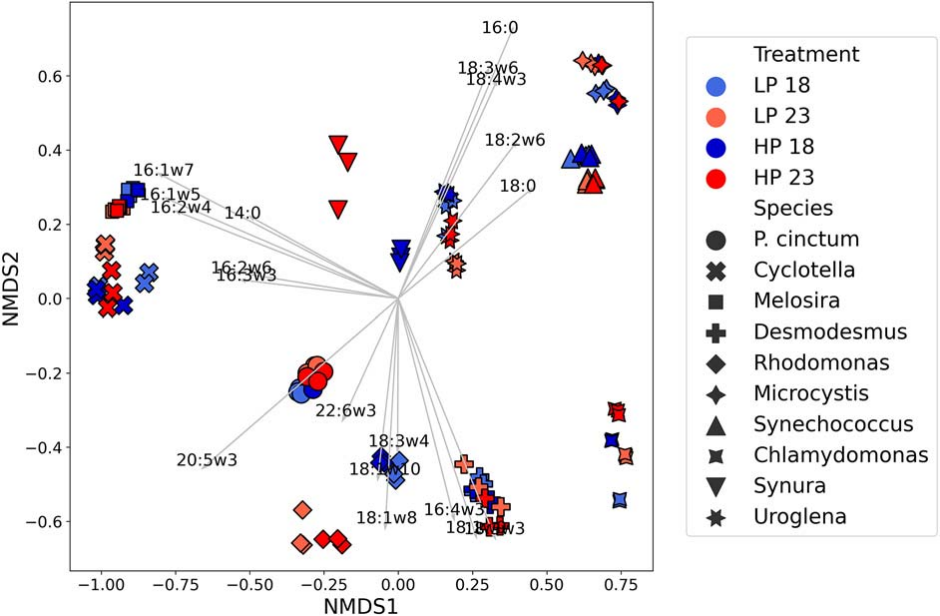
nMDS of fatty acid compositions. Silver arrows indicate fatty acid direction cosines scaled by the square root of their correlation with the axis. Projected fatty acids (structural formulas) represent saturated (14:0, 16:0 and 18:0), monounsaturated (16:1ω7, 16:1ω5, 18:1ω10, 18:1ω9 and 18:1ω8) and polyunsaturated fatty acids [16:2ω6, 16:2ω4, 16:3ω3, 16:4ω3, 18:2ω6, 18:3ω6, 18:3ω3, 18:3ω4, 18:4ω3, 20:5ω3 (EPA) and 22:6ω3 (DHA)]. For a better visualization of each projected fatty acids, please see Fig. S2. Phytoplankton species are representatives of the groups diatoms (*Cyclotella* sp. and *Melosira* sp.), golden algae (*Synura* sp. and *Uroglena* sp.), cyanobacteria (*Microcystis* sp. and *Synechococcus* sp.), green algae (*Chlamydomonas reinhardtii* and *Desmodesmus maximus*), cryptophytes (*Rhodomonas* sp.) and dinoflagellates (*Peridinium cinctum*). Treatment names correspond to culture condition with 18 and 23 denoting temperature in °C and LP and HP denoting phosphorus concentration [0.65 (LP) and 2.58 (HP) μM phosphorus].

Across the studied phytoplankton species, growth rates varied close to one order of magnitude between the slowest (*Rhodomonas*: ⁓0.06 day^−1^) and the fastest (*Uroglena*: ⁓0.30 day^−1^) growing (Fig. 1; Fig. S1). *Synura* showed limited initial growth in LP, and after 2 days of cultivation, cell numbers started to decline (Fig. S1A). Therefore, only *Synura*’s HP treatments (18° and 23°C) were included in the rest of the analysis presented in this study. Phosphorus had a significant effect on growth rate across all species (ANOVA, Table S1), whereas no significant effect was observed for temperature. When including phytoplankton group or species factors in the model, phosphorus affected the growth rate significantly, although it explained only ⁓6% of the variance (ANOVA; Table S1). At the species level, *Melosira* and *Uroglena* were not significantly affected by changes in phosphorus and temperature (Table S1), although positive effect sizes of temperature and phosphorus increases were seen in *Melosira*. Among the significantly affected species, phosphorus explained, on average ⁓42% of the observed variance with the green algae *Chlamydomonas* presenting the highest explained variance (>90%). Temperature explained slightly less variance (⁓39%) in growth rates than phosphorus, and the other studied green algae, *Desmodesmus* had the highest (95%) explained variance. Overall, increases in temperature and phosphorus had a positive effect on growth rate with highly variable effect sizes between species (average Glass Δ > 0 for all treatments comparisons; Fig. 2B).

### Effect of temperature and phosphorus on fatty acids

**Fig. 3:**
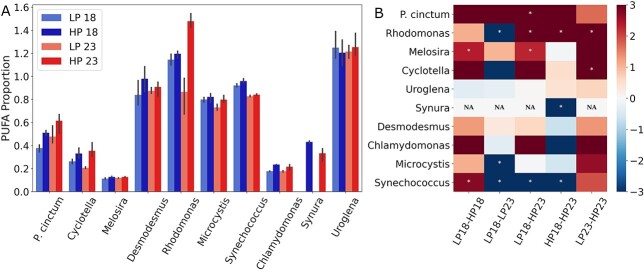
Proportion of polyunsaturated to mono- and saturated fatty acids (**A**) and their normalized changes (Glass′ Δ) between treatments using the first referred treatment as baseline (**B**). Phytoplankton species are representatives of the groups diatoms (*Cyclotella* sp. and *Melosira* sp.), golden algae (*Synura* sp. and *Uroglena* sp.), cyanobacteria (*Microcystis* sp. and *Synechococcus* sp.), green algae (*Chlamydomonas reinhardtii* and *Desmodesmus maximus*), cryptophytes (*Rhodomonas* sp.), and dinoflagellates (*Peridinium cinctum*). Treatment names correspond to culture condition with 18 and 23 denoting temperature in °C and LP and HP denoting phosphorus concentration [0.65 (LP) and 2.58 (HP) μM phosphorus]. * (white marker) denotes statistical difference between treatment comparison in each phytoplankton species.

In total 40 fatty acids were identified and quantified across all 10 phytoplankton species. As expected, the high nutritional value LC-PUFAs EPA and DHA were present in 5 and 6 of the studied phytoplankton species (respectively) corresponding to the species *Melosira*, *Cyclotella* (diatoms), *P. cinctum* (dinoflagellate), *Uroglena*, *Synura* (golden algae) and *Rhodomonas* (cryptophyte) (Table S2). The effect of phosphorus on fatty acid profiles was modest and only significant at the species level, whereas no effect of temperature was observed (Fig. 2; PERMANOVA; Table S3). Since temperature is considered one of the main controllers of fatty acid unsaturation degree, we investigated how the proportion of PUFAs was affected with our treatments. Again, no significant effect of temperature or phosphorus was seen across all phytoplankton or phytoplankton groups (Fig. 3A and B; Table S4). When including the species term in our analysis, phosphorus had a significant effect on PUFA proportion (Table S4). At the species level, *Uroglena* and *Desmodesmus,* and *Chlamydomonas* did not significantly alter their PUFA proportion with changes in temperature or phosphorus (Fig. 3B, Tables S4 and S7). For the rest of the species, temperature explained on average 25%, whereas phosphorus explained 43% of the observed variance. *Synechococcus* was most affected by temperature (⁓90% explained variance), and *Melosira* by phosphorus (⁓64% explained variance). Temperature and phosphorus had contrasting effects on PUFA proportion with increases in phosphorus having an overall positive effect regardless of temperature (average Glass Δ > 0, Fig. 3B), whereas increases in temperature alone led to overall decreases in PUFA proportion (average Glass Δ < 0, Fig. 3B). Nevertheless, all treatments presented large size effect differences between species.

**Fig. 4:**
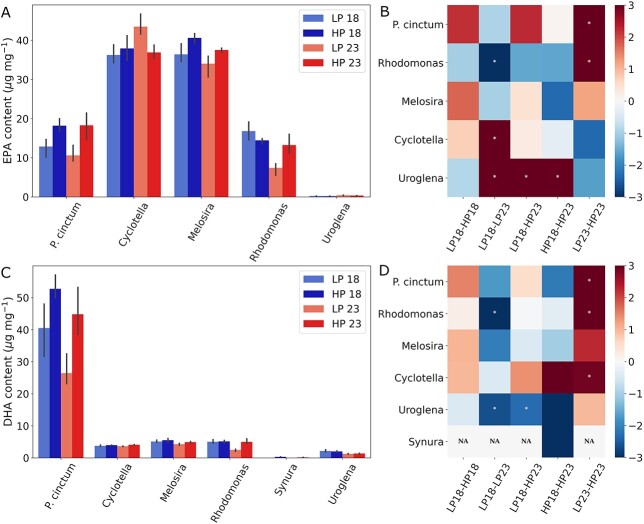
EPA (**A**) and DHA (**C**) content per mg of dry weight and the normalized EPA (**B**) and DHA (**D**) changes (Glass′ Δ) between treatments using the first referred treatment as baseline. Phytoplankton species are representatives of the groups diatoms (*Cyclotella* sp. and *Melosira* sp.), golden algae (*Synura* sp. and *Uroglena* sp.), cryptophytes (*Rhodomonas* sp.) and dinoflagellates (*Peridinium cinctum*). Treatment names correspond to culture condition with 18 and 23 denoting temperature in °C and LP and HP denoting phosphorus concentration [0.65 (LP) and 2.58 (HP) μM phosphorus]. * (white marker) denotes statistical difference between treatment comparison in each phytoplankton species.

In addition to changes in PUFA proportion, we focused on EPA and DHA contents because of their importance for the nutrition of higher trophic levels. Of the studied species that synthesize EPA and DHA, all presented a significant effect of temperature, phosphorus or their interaction (Fig. 4A, Table S5). Both EPA and DHA contents showed large species-specific variation in effect size of temperature and phosphorus (Fig. 4A and B). In terms of EPA content, *P. cinctum* was most affected by changes in phosphorus (69% explained variance; Fig. 4A and B; Table S5), whereas *Uroglena* was most affected by temperature (87% explained variance; Fig. 4A and B; Table S5). EPA content did not present any clear pattern across species to changes in temperature and phosphorus, and the largest average effect size was observed when increasing both temperature and phosphorus (Glass Δ ⁓1.2 ± 2; Fig. 4B). In the case of DHA content, *Cyclotella* was most affected by changes in phosphorus (61% explained variance; Fig. 4B; Table S5), whereas *Uroglena* was most affected by temperature (81% explained variance; Fig. 4B; Table S5). An increase in phosphorus alone led to positive average effect sizes (Glass Δ > 0), whereas the opposite was seen for increases in temperature alone (Glass Δ < 0). The combined effect of temperature and phosphorus was overall negative (Glass Δ ⁓−0.17; Fig. 4B) despite species-specific differences in effect sizes.

**Fig. 5:**
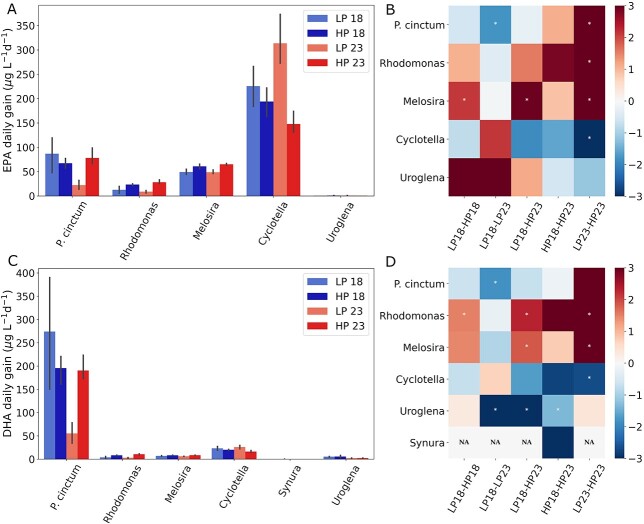
EPA (**A**) and DHA (**C**) production measured as daily gain (μg L^−1^ day^−1^) and the normalized EPA (**B**) and DHA (**D**) production changes (Glass′ Δ) between treatments using the first referred treatment as baseline. Phytoplankton species are representatives of the groups diatoms (*Cyclotella* sp. and *Melosira* sp.), golden algae (*Synura* sp. and *Uroglena* sp.), cryptophytes (*Rhodomonas* sp.) and dinoflagellates (*Peridinium cinctum*). Treatment names correspond to culture condition with 18 and 23 denoting temperature in °C and LP and HP denoting phosphorus concentration [0.65 (LP) and 2.58 (HP) μM phosphorus]. * (white marker) denotes statistical difference between treatment comparison in each phytoplankton species.

The availability of LC-PUFAs in aquatic ecosystems is given by their production, therefore we studied how EPA and DHA daily gain (μg LC-PUFA day^−1^ L^−1^), a proxy for production, is affected by changes in temperature and phosphorus (Fig. 5A and B). The highest EPA gain was observed in *Cyclotella* (220 ± 71 μg EPA L^−1^ day^−1^), and the highest DHA gain in *P. cinctum* (179 ± 99 μg EPA l^−1^ day^−1^). Of our EPA-producing species, *Uroglena’s* daily EPA gain was not significantly affected by changes in temperature or phosphorus (ANOVA, Tables S6 and S7). Within the significantly affected species, temperature explained an average of 6.5% of the observed variance in EPA gain, whereas phosphorus explained 53% of the variance (Table S6). DHA daily gain remained unaffected in *Synura*, whereas the rest of the producing species were significantly affected by changes in temperature or phosphorus (ANOVA, Tables S6 and S7). On average, temperature explained 10% of the observed variance, whereas phosphorus explained 52% of the variance observed in DHA gain across significantly affected species (Table S6). Overall, no uniform effect was observed with treatments for either EPA or DHA gain, and effect sizes varied widely between producing species (Fig. 5B). For both EPA and DHA daily gain, the largest average effect sizes were observed when increasing phosphorus at 23°C (Glass Δ = 2.57 and 3.96 for EPA and DHA, respectively; Fig. 5B). *P. cinctum* and *Cyclotella*, the species with the largest EPA and DHA daily gain (respectively) presented opposing effects to the treatments (Fig. 5A and B). *P. cinctum* was most affected by temperature and had a significant interaction term leading to decreasing DHA daily gain when increasing temperature at low phosphorus, whereas *Cyclotella* was most affected by phosphorus and had a significant interaction term leading to decreasing EPA daily gain when increasing phosphorus at 23°C (Fig. 5; Table S6).

## DISCUSSION

Climate change is expected to alter northern lakes physical and chemical parameters in variety of ways, including higher temperatures and phosphorus (Jennings *et al.*, 2009; Björnerås *et al.*, 2017). Current projections of LC-PUFAs, in particular EPA and DHA, estimate large decreases in the availability of these fatty acids due to higher temperatures (Hixson and Arts, 2016; Colombo *et al.*, 2019). This study provides evidence challenging the aforementioned assumption while supporting the results of Galloway and Winder (2015) regarding the importance of species composition and lake trophic status on the availability of PUFAs. Our growth rate analysis showed an overall positive effect of temperature and phosphorus across the tested phytoplankton species. The strongest effects were observed when increasing both temperature and phosphorus simultaneously. Nevertheless, large species-specific differences in effect sizes were observed to changes in temperature and phosphorus, highlighting how different life histories and plastic changes can modulate phytoplankton responses. Despite that elucidating the mechanisms behind different phytoplankton responses to the studied treatments was not the objective of this study, we believe that phosphorus absorption kinetics and accumulation strategies (Sommer, 1981) combined with changes in optimal growth temperatures (Singh and Singh, 2015) are the main drivers of the observed results. In northern lakes, increases in total phosphorus and temperature are associated with higher frequencies of cyanobacteria blooms (Keva *et al.*, 2020; Vuorio *et al.*, 2020). Of the two tested cyanobacteria, responses to increases in phosphorus and temperature were contrasting, with only *Microcystis* consistently thriving from such changes. This supports observed trends of increased species-specific cyanobacteria blooms under warm and nutrient-rich environments (O’Neil *et al.*, 2012), highlighting that enhanced growth under those conditions is not an overall property of the phytoplankton group. Although our results suggest substantial increases in phytoplankton biomass with climate change, other physical and chemical changes such as reduced light availability driven by increases in dissolved organic carbon concentrations (browning) can also enforce significant pressures in phytoplankton biomass (Taipale *et al.*, 2016; Sullivan *et al.*, 2021), leading to different phytoplankton responses than the ones observed in this study.

When considering the prospects of PUFAs in aquatic ecosystems, temperature increase is associated with PUFA decrease to maintain membrane homeostasis (Sinensky, 1974). In our results, neither temperature nor phosphorus had a generalized effect in phytoplankton fatty acid profiles or the PUFA proportion. At a species level, temperature and phosphorus did affect PUFAs, but variability in the directionality of change and effect size was high. Among the studied species, an increase in phosphorus had an overall positive effect on PUFA proportion. Increases in temperature alone led to an overall decrease in PUFA proportion regardless of initial phosphorus concentration. Altogether, these results suggest that concomitant increases in phosphorus could hinder the decrease in PUFA proportion due to higher temperatures, and that other physical and chemical changes associated with climate change could play a more significant role than previously thought. For example, light availability can modulate cellular levels of PUFAs (Valentine and Valentine, 2004; Wacker *et al.*, 2016) and phytoplankton communities (Bergström *et al.*, 2003; Deininger *et al.*, 2017). Although browning has not been observed to alter PUFA contents in a phytoplankton species common to high dissolved organic carbon lakes (Calderini *et al.*, 2022), overall phytoplankton PUFAs will depend on the interaction between eutrophication, warming and browning. Our results on LC-PUFAs EPA and DHA contents show variable responses of individual phytoplankton species to increases in temperature and phosphorus. Contrary to previous studies (Hixson and Arts, 2016; Colombo *et al.*, 2019), no clear pattern of EPA content to increase in temperature or phosphorus was seen across the studied species. In the case of DHA, a general reduction of DHA content was seen with temperature increases regardless of trophic state, partially agreeing with previous estimates (Colombo *et al.*, 2019). Nevertheless, large species-specific differences in effect size were observed, with some species presenting an increase in DHA content with increases in phosphorus suggesting that concomitant increases in temperature and phosphorus could modulate the negative effect of temperature as we hypothesized. As an extension of these results, it is possible that lakes exhibiting a pattern of warming coupled with declines in nutrient levels (Isles *et al.*, 2018; Isles *et al.*, 2023) could experience reductions of PUFA (particularly DHA) availability.

Although LC-PUFA content is the unit commonly used to study the nutritional quality of seston in lakes, we looked at LC-PUFA production (studied as daily gain) to account for the effect of changes in cell density observed with our treatments. The production of EPA and DHA shows large species-specific differences in directionality and effect size in response to changes in phosphorus and temperature. Of the studied phytoplankton groups, diatoms are considered key EPA producers in aquatic ecosystems, whereas DHA production is associated with dinoflagellates and golden algae (Ahlgren *et al.*, 1992; Galloway and Winder, 2015; Taipale *et al.*, 2016; Jónasdóttir, 2019). Within the studied diatoms, we did not see consistency in their response to changes in temperature and phosphorus, with *Cyclotella* (highest EPA production) showing an overall negative effect to increases in both temperature and phosphorus, whereas *Melosira* showed a substantial increase in EPA production with an increase in phosphorus. Of the studied dinoflagellates and golden algae, both *P. cinctum* (highest DHA daily gain) and *Uroglena* showed an overall decrease of DHA daily gain with increases in temperature. Nevertheless, effect sizes varied largely between these species and within treatments. In summary, the variability observed in effect sizes and directionality of response to warming and phosphorus concentrations across the studied species suggest that species composition will be the determining factor in the availability of PUFAs and LC-PUFAs in lakes as proposed by Galloway and Winder (2015). In addition, processes that increase nutrients (e.g. eutrophication, browning) could play an important role in modulating the effects associated with warming. Altogether, cold water oligotrophic lakes, which commonly present large shares of diatoms in their phytoplankton communities (Taipale *et al.*, 2016; Keva *et al.*, 2020), could present notable fluctuations in EPA production given the observed species-specific responses to environmental change. Meanwhile, DHA production could be more affected in eutrophic lakes with large amounts of dinoflagellates (Taipale *et al.*, 2019). Variations in EPA and DHA production, as a result of species-specific responses of diatoms and dinoflagellates, could potentially alter zooplankton communities due to the high requirements of cladocerans for EPA (Von Elert, 2002) and copepods for DHA (Von Elert and Stampfl, 2000).

Importantly, we did not see a correlation between content and production results for EPA or DHA, showing that these units point to different aspects of phytoplankton LC-PUFAs availability for consumers. Fatty acid content does not account for changes in cell densities when studying the effects of environmental change, hence higher phytoplankton growth can compensate for low fatty acid content. This is especially relevant to consider when extrapolating fatty acid content results to make predictions about how climate change will affect the availability of LC-PUFAs.

## CONCLUSIONS

This study shows that trophic state, as well as phosphorus dynamics, will play a role in the availability of PUFAs and LC-PUFAs in lakes. Both warming and phosphorus will influence PUFAs differently, with temperature driving decreases while phosphorus increases in PUFA availability. EPA and DHA producing species respond differently to increases in temperature and nutrients both in terms of content and daily gain of these fatty acids. Therefore, temperature, phosphorus and phytoplankton composition in lakes will all determine the effects of climate change on the availability of the physiologically essential EPA and DHA.

## Supplementary Material

Supplemental_updated_fbad026

## Data Availability

All data as exportable files and data analysis scripts necessary to replicate the results presented in this study are available at jyx data storage service (10.17011/jyx/dataset/86595). A comprehensive description of the content of each file can be found in the same DOI under the name “Metadata_file.”
