# Supplementary material for: Temperature, phosphorus and species composition will all influence phytoplankton production and content of polyunsaturated fatty acids
Source: J Plankton Res. 2023 Jun 24;45(4):625–35. doi: 10.1093/plankt/fbad026 (PMC10361808; doi:10.1093/plankt/fbad026)
Supplement: Supplemental_updated_fbad026 [file supplemental_updated_fbad026.docx]

**Temperature, phosphorus, and species composition will all influence phytoplankton production and content of polyunsaturated fatty acids**

Marco L. Calderini^1^*, Salli Pääkkönen^2^, Pauliina Salmi^2^, Elina Peltomaa^3^, Sami J. Taipale^1^

_________________________________________________________________________

^1^ Department of Biological and Environmental Science, University of Jyväskylä, Jyväskylä, Finland

^2^ Spectral Imaging Laboratory, Faculty of Information Technology, University of Jyväskylä, Jyväskylä, Finland

^3^ Department of Forest Sciences, University of Helsinki, Helsinki, Finland

*Correspondence: email: marco.92.calderini@jyu.fi

Present address: University of Jyvaskyla, Department of Biological and Environmental Science, P.O. Box 35, FI-40014 University of Jyvaskyla, Finland.

*Corresponding author

*Present address: University of Jyväskylä, Department of Biological and Environmental Science, P.O. Box 35, FI-40014 University of Jyväskylä, Finland.

**E-mail addresses and ORCID*:

-marco.92.calderini@jyu.fi

-https://orcid.org/0000-0003-2532-3167


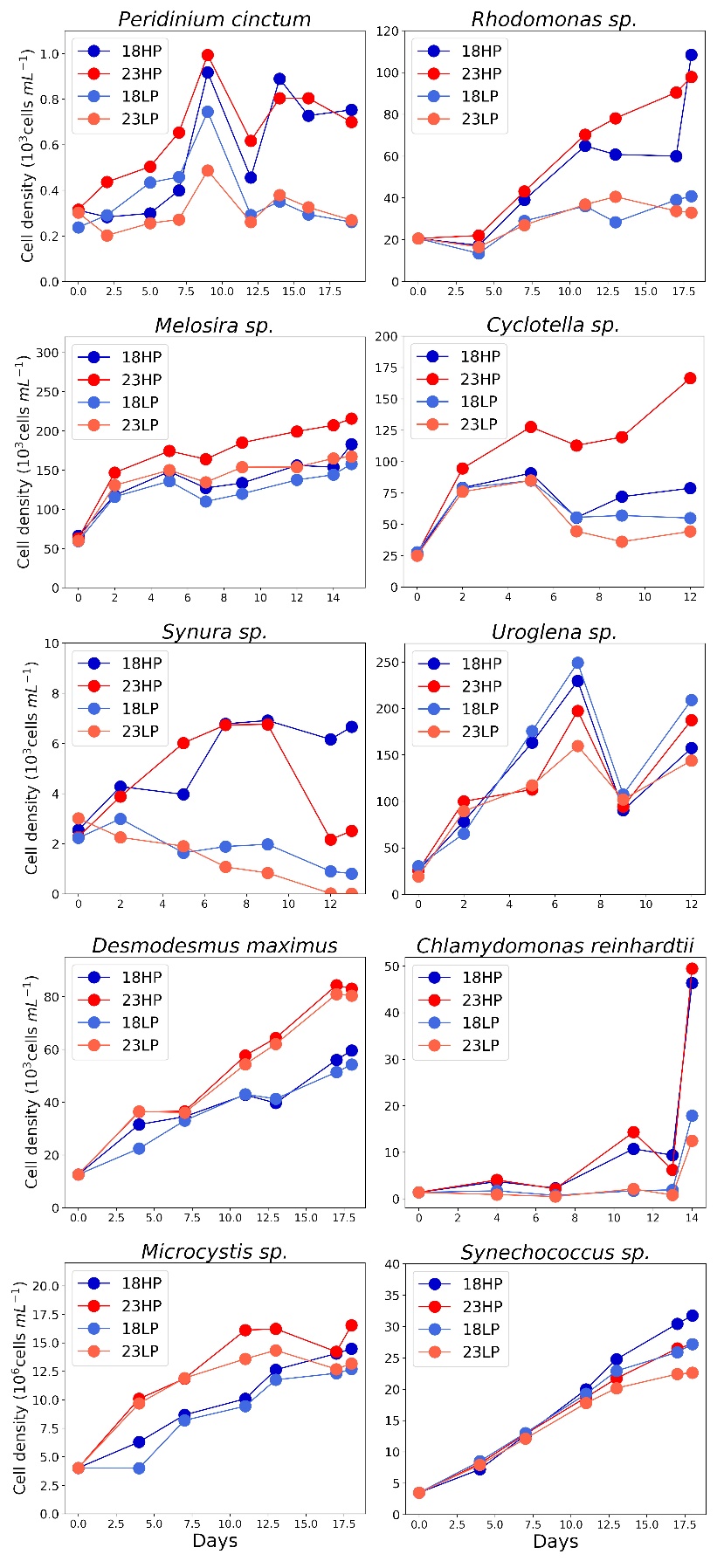


**Fig. S1.** Changes in cell number (cell mL^−1^) of the studied freshwater phytoplankton species during their respective cultivation time (days). Phytoplankton species are representatives of the groups diatoms (*Cyclotella* sp. and *Melosira* sp.), chrysophytes (*Synura* sp. and *Uroglena* sp.), cyanobacteria (*Microcystis* sp. and *Synechococcus* sp.), green algae (*Chlamydomonas reinhardtii* and *Desmodesmus maximus*), cryptophytes (*Rhodomonas* sp.), and dinoflagellates (*Peridinium cinctum*). Treatment names correspond to culture condition with 18 and 23 denoting temperature in °C and LP and HP denoting phosphorus concentration (0.65 [LP] and 2.58 [HP] µM phosphorus).


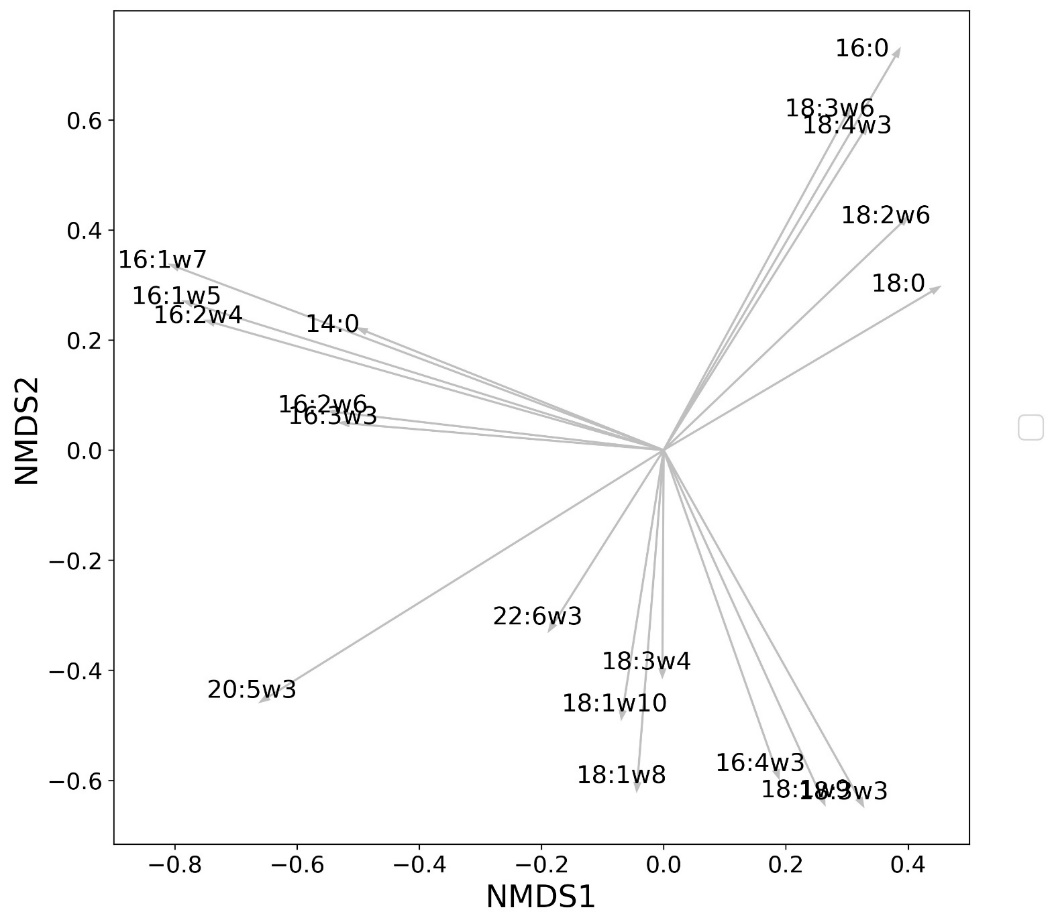


**Figure S2 Fatty acid projections of Non-metric multidimentional scaling (nMDS) results.** Silver arrows indicate fatty acid direction cosines scaled by the square root of their correlation with the axis. Projected fatty acids represent saturated (14:0, 16:0 and 18:0), monounsaturated (16:1ω7, 16:1ω5, 18:1ω10, 18:1ω9 and 18:1ω8) and polyusaturated fatty acids (16:2ω6, 16:2ω4, 16:3ω3, 16:4ω3, 18:2ω6, 18:3ω6, 18:3ω3, 18:3ω4, 18:4ω3, 20:5ω3 [EPA] and 22:6ω3 [DHA].

**Table S1 Growth rates ANOVA results.** Factors: T= temperature, P= phosphorus, T:P= interaction between temperature and phosphorus. Df= degrees of freedom, Sq= sum of squares. In bold p-values <0.05. All species analysis do not include *Synura* sp. due to the collapse of low phosphorus cultures.

| **ANOVA** | **Factor** | **Df** | **Sum Sq** | **Mean Sq** | **F value** | **p-value** | **Explained variance** |
| --- | --- | --- | --- | --- | --- | --- | --- |
| All species | T | 1 | 0.0000 | 0.0000 | 0 | 0.98 | 0.0 |
|  | P | 1 | 0.0700 | 0.0700 | 7.541 | **0.01** | 6.1 |
|  | T:P | 1 | 0.0052 | 0.0052 | 0.563 | 0.45 | 0.5 |
|  | Residuals | 116 | 1.0774 | 0.0093 |  |  | 93.5 |
| All species | Group | 5 | 0.1460 | 0.0292 | 3.481 | **0.01** | 12.7 |
|  | T | 1 | 0.0000 | 0.0000 | 0 | 0.98 | 0.0 |
|  | P | 1 | 0.0700 | 0.0700 | 8.348 | **<0.01** | 6.1 |
|  | T:P | 1 | 0.0052 | 0.0052 | 0.624 | 0.43 | 0.5 |
|  | Residuals | 111 | 0.9314 | 0.0084 |  |  | 80.8 |
| All species | Species | 9 | 0.8793 | 0.0977 | 52.775 | **<0.01** | 76.29 |
|  | T | 1 | 0.0000 | 0.0000 | 0.002 | 0.97 | 0.00 |
|  | P | 1 | 0.0700 | 0.0700 | 37.836 | **<0.01** | 6.07 |
|  | T:P | 1 | 0.0052 | 0.0052 | 2.827 | 0.10 | 0.45 |
|  | Residuals | 107 | 0.1981 | 0.0019 |  |  | 17.19 |
| *P. cinctum* | T | 1 | 0.0032 | 0.0032 | 5.7 | **0.04** | 19.9 |
|  | P | 1 | 0.0032 | 0.0032 | 5.816 | **0.04** | 20.3 |
|  | T:P | 1 | 0.0050 | 0.0050 | 9.069 | **0.02** | 31.7 |
|  | Residuals | 8 | 0.0044 | 0.0006 |  |  | 28 |
| *Rhodomonas* | T | 1 | 0.0019 | 0.0019 | 6.46 | **0.03** | 13.4 |
|  | P | 1 | 0.0096 | 0.0096 | 33.47 | **<0.01** | 69.4 |
|  | T:P | 1 | 0.0001 | 0.0001 | 0.309 | 0.59 | 0.6 |
|  | Residuals | 8 | 0.0023 | 0.0003 |  |  | 16.6 |
| *Cyclotella* | T | 1 | 0.0054 | 0.0054 | 22.659 | **<0.01** | 33.5 |
|  | P | 1 | 0.0076 | 0.0076 | 31.822 | **<0.01** | 47 |
|  | T:P | 1 | 0.0012 | 0.0012 | 5.246 | **0.05** | 7.7 |
|  | Residuals | 8 | 0.0019 | 0.0002 |  |  | 11.8 |
| *Melosira* | T | 1 | 0.0002 | 0.0002 | 4.463 | 0.07 | 23.9 |
|  | P | 1 | 0.0002 | 0.0002 | 4.293 | 0.07 | 23 |
|  | T:P | 1 | 0.0001 | 0.0001 | 1.889 | 0.21 | 10.1 |
|  | Residuals | 8 | 0.0004 | 0.0000 |  |  | 42.9 |
| *Uroglena* | T | 1 | 0.0005 | 0.0005 | 0.403 | 0.54 | 4.5 |
|  | P | 1 | 0.0000 | 0.0000 | 0.002 | 0.96 | 0 |
|  | T:P | 1 | 0.0006 | 0.0006 | 0.555 | 0.48 | 6.2 |
|  | Residuals | 8 | 0.0091 | 0.0011 |  |  | 89.3 |
| *Synura* | T | 1 | 0.0005 | 0.0005 | 1.229 | 0.33 | 23.508 |
|  | Residuals | 4 | 0.0016 | 0.0004 |  |  | 76.492 |
| *Chlamidomonas* | T | 1 | 0.0003 | 0.0003 | 8.068 | **0.02** | 1.4 |
|  | P | 1 | 0.0209 | 0.0209 | 526.18 | **<0.01** | 94.1 |
|  | T:P | 1 | 0.0007 | 0.0007 | 16.919 | **<0.01** | 3 |
|  | Residuals | 8 | 0.0003 | 0.0000 |  |  | 1.4 |
| *Desmodesmus* | T | 1 | 0.0019 | 0.0019 | 249.43 | **<0.01** | 94.7 |
|  | P | 1 | 0.0000 | 0.0000 | 5.389 | **0.05** | 2 |
|  | T:P | 1 | 0.0000 | 0.0000 | 0.629 | 0.45 | 0.2 |
|  | Residuals | 8 | 0.0001 | 0.0000 |  |  | 3 |
| *Microcystis* | T | 1 | 0.0009 | 0.0009 | 71.547 | **<0.01** | 76.2 |
|  | P | 1 | 0.0002 | 0.0002 | 13.488 | **0.01** | 14.4 |
|  | T:P | 1 | 0.0000 | 0.0000 | 0.886 | 0.37 | 0.9 |
|  | Residuals | 8 | 0.0001 | 0.0000 |  |  | 8.5 |
| *Synechococcus* | T | 1 | 0.0002 | 0.0002 | 15.484 | **<0.01** | 34.6 |
|  | P | 1 | 0.0003 | 0.0003 | 21.312 | **<0.01** | 47.6 |
|  | T:P | 1 | 0.0000 | 0.0000 | 0.009 | 0.93 | 0 |
|  | Residuals | 8 | 0.0001 | 0.0000 |  |  | 17.9 |

**Table S2 Average fatty acid content (µg FA mg^-1^) of the studied phytoplankton species at each tested temperature (Tem: 18 and 23 °C) and phosphorus concentration (P: 0.65 [LP] and 2.58 [HP] µM phosphorus).** The presented fatty acids have a contribution >1% of the total fatty of at least one of the studied species. To see the full list of identified and quantified fatty acids, please see jyx data storage (https://doi.org/10.17011/jyx/dataset/86595).

| **Species** | **P** | **Tem** | **14:0** | **16:0** | **16:1w7** | **16:1w5** | **16:2w6** | **16:2w4** | **16:3w3** | **18:00** | **16:4w3** | **18:1w10** | **18:1w9** | **18:1w8** | **18:1w7** | **18:2w6** | **18:3w6** | **18:3w3** | **18:3w4** | **18:4w3** | **18:5w3** | **20:4w6** | **20:5w3** | **22:5w6** | **22:6w3** |
| --- | --- | --- | --- | --- | --- | --- | --- | --- | --- | --- | --- | --- | --- | --- | --- | --- | --- | --- | --- | --- | --- | --- | --- | --- | --- |
| *Chlamydomonas* | HP | 18 | 2.45 | 109.04 | 0.71 | 0.00 | 2.65 | 0.53 | 0.00 | 4.04 | 19.26 | 0.00 | 310.49 | 0.00 | 44.34 | 30.80 | 0.00 | 50.14 | 0.00 | 3.69 | 0.00 | 0.00 | 0.00 | 0.00 | 0.00 |
| *Chlamydomonas* | HP | 23 | 2.59 | 149.23 | 0.84 | 0.00 | 3.58 | 0.61 | 0.00 | 5.62 | 20.96 | 0.00 | 369.37 | 0.00 | 49.30 | 41.31 | 0.00 | 52.61 | 0.00 | 3.27 | 0.00 | 0.00 | 0.00 | 0.00 | 0.00 |
| *Chlamydomonas* | LP | 18 | 3.02 | 142.87 | 0.98 | 0.00 | 2.15 | 0.46 | 0.00 | 6.05 | 23.43 | 0.00 | 512.62 | 0.00 | 61.28 | 34.61 | 0.00 | 60.08 | 0.00 | 4.08 | 0.00 | 0.00 | 0.00 | 0.00 | 0.00 |
| *Chlamydomonas* | LP | 23 | 3.34 | 178.33 | 0.66 | 0.00 | 3.16 | 0.60 | 0.00 | 8.16 | 22.86 | 0.00 | 515.31 | 0.00 | 59.07 | 42.59 | 0.00 | 58.97 | 0.00 | 3.46 | 0.00 | 0.00 | 0.00 | 0.00 | 0.00 |
| *Cyclotella* | HP | 18 | 34.42 | 48.08 | 139.74 | 4.47 | 2.24 | 6.33 | 19.83 | 1.04 | 0.00 | 0.00 | 2.04 | 0.00 | 4.10 | 0.80 | 0.36 | 0.00 | 0.40 | 4.56 | 0.00 | 0.00 | 37.90 | 0.00 | 3.97 |
| *Cyclotella* | HP | 23 | 34.28 | 55.84 | 136.30 | 3.95 | 3.96 | 6.83 | 24.34 | 0.86 | 0.00 | 0.00 | 1.05 | 0.00 | 4.57 | 0.75 | 0.29 | 0.00 | 0.94 | 3.02 | 0.00 | 0.00 | 36.88 | 0.00 | 4.06 |
| *Cyclotella* | LP | 18 | 37.27 | 57.71 | 160.92 | 4.70 | 2.11 | 5.76 | 17.76 | 1.48 | 0.00 | 0.00 | 6.36 | 0.00 | 5.69 | 0.68 | 0.38 | 0.00 | 0.35 | 4.78 | 0.00 | 0.00 | 36.24 | 0.00 | 3.73 |
| *Cyclotella* | LP | 23 | 50.82 | 84.05 | 246.87 | 6.42 | 3.50 | 5.81 | 17.86 | 1.20 | 0.00 | 0.00 | 1.54 | 0.00 | 3.38 | 1.04 | 0.61 | 0.00 | 0.48 | 4.69 | 0.00 | 0.00 | 43.47 | 0.00 | 3.62 |
| *Desmodesmus* | HP | 18 | 0.65 | 1.42 | 0.35 | 0.00 | 0.00 | 0.01 | 0.00 | 0.30 | 1.30 | 0.00 | 1.58 | 0.42 | 0.00 | 0.29 | 0.00 | 3.41 | 0.16 | 0.00 | 0.00 | 0.00 | 0.00 | 0.00 | 0.00 |
| *Desmodesmus* | HP | 23 | 0.58 | 1.35 | 0.27 | 0.00 | 0.00 | 0.02 | 0.00 | 0.29 | 1.14 | 0.00 | 1.59 | 0.57 | 0.00 | 0.26 | 0.00 | 3.16 | 0.21 | 0.00 | 0.00 | 0.00 | 0.00 | 0.00 | 0.00 |
| *Desmodesmus* | LP | 18 | 0.75 | 1.47 | 0.33 | 0.00 | 0.00 | 0.02 | 0.00 | 0.42 | 1.14 | 0.00 | 1.90 | 0.44 | 0.00 | 0.28 | 0.00 | 3.25 | 0.15 | 0.00 | 0.00 | 0.00 | 0.00 | 0.00 | 0.00 |
| *Desmodesmus* | LP | 23 | 0.53 | 1.38 | 0.29 | 0.00 | 0.00 | 0.02 | 0.00 | 0.33 | 1.06 | 0.00 | 1.40 | 0.47 | 0.00 | 0.23 | 0.00 | 2.89 | 0.16 | 0.00 | 0.00 | 0.00 | 0.00 | 0.00 | 0.00 |
| *Melosira* | HP | 18 | 71.24 | 124.70 | 317.96 | 7.15 | 2.21 | 7.02 | 1.29 | 0.87 | 0.00 | 0.00 | 1.06 | 0.00 | 2.86 | 0.89 | 0.57 | 0.43 | 0.43 | 7.18 | 0.00 | 0.00 | 40.60 | 0.00 | 5.50 |
| *Melosira* | HP | 23 | 65.60 | 110.69 | 311.84 | 6.64 | 3.24 | 6.75 | 1.84 | 1.21 | 0.00 | 0.00 | 1.43 | 0.00 | 3.32 | 1.35 | 0.53 | 0.53 | 0.64 | 4.73 | 0.00 | 0.00 | 37.51 | 0.00 | 4.92 |
| *Melosira* | LP | 18 | 68.77 | 126.10 | 333.71 | 6.72 | 1.86 | 6.31 | 1.14 | 1.19 | 0.00 | 0.00 | 1.23 | 0.00 | 3.07 | 1.12 | 0.62 | 0.57 | 0.44 | 6.52 | 0.00 | 0.00 | 36.38 | 0.00 | 5.10 |
| *Melosira* | LP | 23 | 60.05 | 103.08 | 306.77 | 6.11 | 2.80 | 5.38 | 1.57 | 1.54 | 0.00 | 0.00 | 1.71 | 0.00 | 3.75 | 2.06 | 0.67 | 0.87 | 0.49 | 4.09 | 0.00 | 0.00 | 34.00 | 0.00 | 4.27 |
| *Microcystis* | HP | 18 | 0.65 | 31.47 | 0.79 | 0.00 | 0.00 | 0.00 | 0.00 | 2.54 | 0.00 | 0.00 | 0.51 | 0.00 | 0.72 | 7.69 | 11.89 | 4.23 | 0.00 | 5.93 | 0.00 | 0.11 | 0.00 | 0.00 | 0.00 |
| *Microcystis* | HP | 23 | 0.64 | 32.65 | 0.80 | 0.00 | 0.00 | 0.00 | 0.00 | 2.49 | 0.00 | 0.00 | 0.65 | 0.00 | 0.84 | 8.68 | 12.37 | 3.84 | 0.00 | 5.32 | 0.00 | 0.10 | 0.00 | 0.00 | 0.00 |
| *Microcystis* | LP | 18 | 1.02 | 32.99 | 0.93 | 0.00 | 0.00 | 0.00 | 0.00 | 2.56 | 0.00 | 0.00 | 0.43 | 0.00 | 0.64 | 6.40 | 12.21 | 4.37 | 0.00 | 7.56 | 0.00 | 0.12 | 0.00 | 0.00 | 0.00 |
| *Microcystis* | LP | 23 | 0.82 | 30.87 | 0.85 | 0.00 | 0.00 | 0.00 | 0.00 | 2.50 | 0.00 | 0.00 | 0.78 | 0.00 | 1.33 | 9.58 | 10.98 | 2.58 | 0.00 | 3.69 | 0.00 | 0.09 | 0.00 | 0.00 | 0.00 |
| *P. cinctum* | HP | 18 | 26.26 | 49.59 | 14.87 | 0.00 | 0.00 | 0.00 | 0.00 | 5.05 | 0.00 | 0.00 | 93.15 | 1.69 | 0.00 | 1.94 | 0.00 | 5.91 | 0.00 | 9.83 | 9.30 | 0.00 | 18.17 | 0.00 | 52.76 |
| *P. cinctum* | HP | 23 | 19.55 | 40.00 | 13.31 | 0.00 | 0.00 | 0.00 | 0.00 | 3.66 | 0.00 | 0.00 | 60.95 | 2.79 | 0.00 | 0.81 | 0.00 | 4.51 | 0.00 | 11.09 | 7.82 | 0.00 | 18.28 | 0.00 | 44.79 |
| *P. cinctum* | LP | 18 | 23.98 | 45.32 | 20.83 | 0.00 | 0.00 | 0.00 | 0.00 | 4.18 | 0.00 | 0.00 | 89.39 | 6.05 | 0.00 | 1.97 | 0.00 | 4.35 | 0.00 | 6.63 | 6.39 | 0.00 | 12.85 | 0.00 | 40.50 |
| *P. cinctum* | LP | 23 | 15.00 | 30.35 | 10.94 | 0.00 | 0.00 | 0.00 | 0.00 | 2.16 | 0.00 | 0.00 | 42.83 | 3.95 | 0.00 | 0.65 | 0.00 | 3.19 | 0.00 | 5.83 | 4.29 | 0.00 | 10.61 | 0.00 | 26.44 |
| *Rhodomonas* | HP | 18 | 9.95 | 22.84 | 1.41 | 0.13 | 0.00 | 0.00 | 0.00 | 0.95 | 0.06 | 2.17 | 10.75 | 9.81 | 0.16 | 1.59 | 0.05 | 28.60 | 18.54 | 0.00 | 0.00 | 0.15 | 14.43 | 2.52 | 5.13 |
| *Rhodomonas* | HP | 23 | 6.05 | 12.37 | 1.27 | 0.22 | 0.00 | 0.00 | 0.00 | 0.52 | 0.15 | 2.48 | 4.24 | 9.60 | 0.12 | 0.99 | 0.04 | 19.94 | 13.46 | 0.00 | 0.00 | 0.12 | 13.23 | 3.08 | 4.99 |
| *Rhodomonas* | LP | 18 | 9.57 | 27.22 | 1.76 | 0.19 | 0.00 | 0.00 | 0.00 | 1.82 | 0.09 | 2.80 | 14.98 | 14.05 | 0.27 | 1.95 | 0.06 | 34.61 | 22.82 | 0.00 | 0.00 | 0.19 | 16.77 | 3.32 | 5.01 |
| *Rhodomonas* | LP | 23 | 3.46 | 7.07 | 1.71 | 0.24 | 0.00 | 0.00 | 0.00 | 0.60 | 0.30 | 2.05 | 1.90 | 12.75 | 0.24 | 0.42 | 0.03 | 6.79 | 6.27 | 0.00 | 0.00 | 0.11 | 7.42 | 2.66 | 2.48 |
| *Synechococcus* | HP | 18 | 1.06 | 31.42 | 2.01 | 0.00 | 0.00 | 0.00 | 0.00 | 1.53 | 0.00 | 0.00 | 0.89 | 0.00 | 1.46 | 1.70 | 0.18 | 8.91 | 0.00 | 26.06 | 0.00 | 0.00 | 0.00 | 0.00 | 0.00 |
| *Synechococcus* | HP | 23 | 1.79 | 58.21 | 2.90 | 0.00 | 0.00 | 0.00 | 0.00 | 2.65 | 0.00 | 0.00 | 2.09 | 0.00 | 4.50 | 5.50 | 0.79 | 15.67 | 0.00 | 38.61 | 0.00 | 0.00 | 0.00 | 0.00 | 0.00 |
| *Synechococcus* | LP | 18 | 1.11 | 33.61 | 2.44 | 0.00 | 0.00 | 0.00 | 0.00 | 1.60 | 0.00 | 0.00 | 0.96 | 0.00 | 2.32 | 1.80 | 0.18 | 9.57 | 0.00 | 27.17 | 0.00 | 0.00 | 0.00 | 0.00 | 0.00 |
| *Synechococcus* | LP | 23 | 1.14 | 29.55 | 1.55 | 0.00 | 0.00 | 0.00 | 0.00 | 1.24 | 0.00 | 0.00 | 1.05 | 0.00 | 2.25 | 2.41 | 0.37 | 8.07 | 0.00 | 19.63 | 0.00 | 0.00 | 0.00 | 0.00 | 0.00 |
| *Synura* | HP | 18 | 6.22 | 7.05 | 2.60 | 0.00 | 0.00 | 0.00 | 0.00 | 1.44 | 0.00 | 0.00 | 4.22 | 0.00 | 10.82 | 0.00 | 0.66 | 3.14 | 0.00 | 5.11 | 0.00 | 0.00 | 0.00 | 2.51 | 0.31 |
| *Synura* | HP | 23 | 4.49 | 5.88 | 2.32 | 0.00 | 0.00 | 0.00 | 0.00 | 1.17 | 0.00 | 0.00 | 2.14 | 0.00 | 11.69 | 0.00 | 0.27 | 1.19 | 0.00 | 2.16 | 0.00 | 0.00 | 0.00 | 3.38 | 0.14 |
| *Uroglena* | HP | 18 | 18.56 | 9.78 | 1.51 | 0.00 | 0.00 | 0.00 | 0.00 | 0.47 | 0.00 | 0.00 | 1.82 | 0.00 | 1.55 | 14.41 | 1.52 | 6.94 | 5.37 | 5.37 | 0.00 | 0.30 | 0.18 | 4.57 | 2.00 |
| *Uroglena* | HP | 23 | 20.30 | 11.95 | 2.01 | 0.00 | 0.00 | 0.00 | 0.00 | 0.69 | 0.00 | 0.00 | 4.19 | 0.00 | 1.34 | 18.83 | 1.62 | 8.76 | 6.92 | 6.92 | 0.00 | 0.46 | 0.35 | 5.23 | 1.35 |
| *Uroglena* | LP | 18 | 20.55 | 11.91 | 1.81 | 0.00 | 0.00 | 0.00 | 0.00 | 0.66 | 0.00 | 0.00 | 2.93 | 0.00 | 1.69 | 18.26 | 1.70 | 8.16 | 6.97 | 6.97 | 0.00 | 0.25 | 0.21 | 4.73 | 2.16 |
| *Uroglena* | LP | 23 | 22.35 | 15.62 | 2.13 | 0.00 | 0.00 | 0.00 | 0.00 | 1.05 | 0.00 | 0.00 | 8.34 | 0.00 | 1.52 | 25.33 | 1.80 | 10.44 | 8.20 | 8.20 | 0.00 | 0.65 | 0.42 | 5.18 | 1.23 |

**Table S3 PERMANOVA results using fatty acid contribution data.** Factors: T= temperature, P= phosphorus, T:P= interaction between temperature and phosphorus. Df= degrees of freedom, Sq= sum of squares. In bold p-values <0.05. All species analysis do not include *Synura* sp. due to the collapse of low phosphorus cultures.

| **ANOVA** | **Factor** | **Df** | **Sum Sq** | **R2** | **F value** | **p-value** | **Explained variance** |
| --- | --- | --- | --- | --- | --- | --- | --- |
| All species | P | 1 | 0.0113 | 0.00056 | 0.058 | 1 | 0.0 |
|  | T | 1 | -0.0016 | -0.00008 | -0.0081 | 1 | 0.0 |
|  | P:T | 1 | 0.0176 | 0.00087 | 0.0905 | 1.00 | 0.0 |
|  | Residual | 104 | 20.2759 | 0.99865 |  |  | 1.0 |
| All species | P | 1 | 0.0113 | 0.00056 | 0.3821 | 0.85 | 0.00 |
|  | T | 1 | -0.0016 | -0.00008 | -0.053 | 1 | 0.00 |
|  | Group | 5 | 17.3561 | 0.85484 | 117.2876 | **<0.01** | 0.85 |
|  | P:T | 1 | 0.0075 | 0.00037 | 0.2526 | 0.94 | 0.00 |
|  | Residual | 99 | 2.93 | 0.14431 |  |  | 0.14 |
| All species | P | 1 | 0.0113 | 0.00056 | 3.4832 | **<0.01** | 0.00 |
|  | T | 1 | -0.0016 | -0.00008 | -0.4835 | 1 | 0.00 |
|  | Species | 9 | 19.9771 | 0.98393 | 769.0871 | **<0.01** | 0.98 |
|  | P:T | 1 | 0.0048 | 0.00024 | 1.4715 | 0.21 | 0.00 |
|  | Residual | 107 | 0.3117 | 0.01535 |  |  | 0.02 |

**Table S4 Polyunsaturated fatty acid (PUFA) proportion ANOVA results.** Factors: T= temperature, P= phosphorus, T:P= interaction between temperature and phosphorus. Df= degrees of freedom, Sq= sum of squares. In bold p-values <0.05. All species analysis do not include *Synura* sp. due to the collapse of low phosphorus cultures.

| **ANOVA** | **Factor** | **Df** | Sum Sq | Mean Sq | F value | **p-value** | **Explained variance** |
| --- | --- | --- | --- | --- | --- | --- | --- |
| All species | T | 1 | 0.002 | 0.00185 | 0.011 | 0.92 | 0.01 |
|  | P | 1 | 0.133 | 0.13329 | 0.797 | 0.37 | 0.76 |
|  | T:P | 1 | 0.008 | 0.00752 | 0.045 | 0.83 | 0.05 |
|  | Residuals | 104 | 17.389 | 0.1672 |  |  | 99.18 |
| All species | Group | 5 | 13.342 | 2.6684 | 65.649 | **<0.01** | 76.11 |
|  | T | 1 | 0.002 | 0.0018 | 0.045 | 0.83 | 0.01 |
|  | P | 1 | 0.151 | 0.1514 | 3.726 | 0.06 | 0.86 |
|  | T:P | 1 | 0.012 | 0.0123 | 0.304 | 0.58 | 0.07 |
|  | Residuals | 99 | 4.024 | 0.0406 |  |  | 22.95 |
| All species | Species | 8 | 16.471 | 2.0589 | 241.578 | **<0.01** | 93.95 |
|  | T | 1 | 0.002 | 0.0018 | 0.217 | 0.64 | 0.01 |
|  | P | 1 | 0.208 | 0.2084 | 24.451 | **<0.01** | 1.19 |
|  | T:P | 1 | 0.032 | 0.0321 | 3.763 | 0.06 | 0.18 |
|  | Residuals | 96 | 0.818 | 0.0085 |  |  | 4.67 |
| *P. cinctum* | T | 1 | 0.03135 | 0.03135 | 7.541 | **0.03** | 26.3 |
|  | P | 1 | 0.0545 | 0.0545 | 13.109 | **0.01** | 45.7 |
|  | T:P | 1 | 0.00002 | 0.00002 | 0.004 | 0.95 | 0 |
|  | Residuals | 8 | 0.03326 | 0.00416 |  |  | 27.9 |
| *Rhodomonas* | T | 1 | 0 | 0 | 0.001 | 0.98 | 0 |
|  | P | 1 | 0.3306 | 0.3306 | 37.765 | **<0.01** | 51.9 |
|  | T:P | 1 | 0.2368 | 0.2368 | 27.048 | **<0.01** | 37.2 |
|  | Residuals | 8 | 0.07 | 0.0088 |  |  | 11 |
| *Cyclotella* | T | 1 | 0.00079 | 0.00079 | 0.404 | 0.54 | 1.4 |
|  | P | 1 | 0.03542 | 0.03542 | 18.227 | **<0.01** | 62.7 |
|  | T:P | 1 | 0.00469 | 0.00469 | 2.416 | 0.16 | 8.3 |
|  | Residuals | 8 | 0.01555 | 0.00194 |  |  | 27.5 |
| *Melosira* | T | 1 | 2.71E-05 | 2.71E-05 | 1.36 | 0.28 | 4.4 |
|  | P | 1 | 0.00039 | 3.88E-04 | 19.468 | **<0.01** | 63.6 |
|  | T:P | 1 | 3.55E-05 | 3.55E-05 | 1.781 | 0.22 | 5.8 |
|  | Residuals | 8 | 0.00016 | 1.99E-05 |  |  | 26.1 |
| *Uroglena* | T | 1 | 0.00015 | 0.000149 | 0.014 | 0.91 | 0.2 |
|  | P | 1 | 0.00003 | 0.000028 | 0.003 | 0.96 | 0.0 |
|  | T:P | 1 | 0.00483 | 0.004831 | 0.465 | 0.52 | 5.5 |
|  | Residuals | 8 | 0.0831 | 0.010388 |  |  | 94.3 |
| *Desmodesmus* | T | 1 | 0.00096 | 0.00097 | 0.133 | 0.73 | 1.1 |
|  | P | 1 | 0.02299 | 0.02299 | 3.169 | 0.11 | 25.4 |
|  | T:P | 1 | 0.0084 | 0.0084 | 1.158 | 0.31 | 9.3 |
|  | Residuals | 8 | 0.05803 | 0.00725 |  |  | 64.2 |
| *Microcystis* | T | 1 | 0.00647 | 0.00647 | 6.673 | **0.03** | 29.6 |
|  | P | 1 | 0.00609 | 0.00609 | 6.283 | **0.04** | 27.9 |
|  | T:P | 1 | 0.00154 | 0.00154 | 1.593 | 0.24 | 7.1 |
|  | Residuals | 8 | 0.00775 | 0.00097 |  |  | 35.5 |
| *Synechococcus* | T | 1 | 0.03318 | 0.03318 | 196.536 | **<0.01** | 89.7 |
|  | P | 1 | 0.00198 | 0.00198 | 11.716 | **0.01** | 5.4 |
|  | T:P | 1 | 0.00046 | 0.00046 | 2.719 | 0.14 | 1.2 |
|  | Residuals | 8 | 0.00135 | 0.00017 |  |  | 3.7 |

**Table S5 EPA and DHA fatty acid content (ng FA mg^-1^) ANOVA results.** Factors: T= temperature, P= phosphorus, T:P= interaction between temperature and phosphorus. Df= degrees of freedom, Sq= sum of squares. In bold p-values <0.05.

| **ANOVA** | **Fatty acid** | **Factor** | **Df** | **Sum Sq** | **Mean Sq** | **F value** | **p-value** | **Explained variance** |
| --- | --- | --- | --- | --- | --- | --- | --- | --- |
| *P. cinctum* | EPA | T | 1 | 3388305 | 3388305 | 0.538 | 0.48 | 1.8 |
|  |  | P | 1 | 1.27E+08 | 1.27E+08 | 20.125 | **<0.01** | 68.6 |
|  |  | T:P | 1 | 4139833 | 4139833 | 0.658 | 0.44 | 2.2 |
|  |  | Residuals | 8 | 50352167 | 6294021 |  |  | 27.3 |
|  | DHA | T | 1 | 3.64E+08 | 3.64E+08 | 8.748 | **0.02** | 25.5 |
|  |  | P | 1 | 7.03E+08 | 7.03E+08 | 16.891 | **<0.01** | 49.2 |
|  |  | T:P | 1 | 27901599 | 27901599 | 0.671 | 0.44 | 2 |
|  |  | Residuals | 8 | 3.33E+08 | 41602957 |  |  | 23.3 |
| *Rhodomonas* | EPA | T | 1 | 83504594 | 83504594 | 22.797 | **<0.01** | 48.7 |
|  |  | P | 1 | 9036472 | 9036472 | 2.467 | 0.15 | 5.3 |
|  |  | T:P | 1 | 49763780 | 49763780 | 13.586 | **0.01** | 29 |
|  |  | Residuals | 8 | 29304008 | 3663001 |  |  | 17.1 |
|  | DHA | T | 1 | 5345058 | 5345058 | 15.7 | **<0.01** | 30.4 |
|  |  | P | 1 | 5220081 | 5220081 | 15.33 | **<0.01** | 29.7 |
|  |  | T:P | 1 | 4278831 | 4278831 | 12.56 | **0.01** | 24.4 |
|  |  | Residuals | 8 | 2724233 | 340529 |  |  | 15.5 |
| *Melosira* | EPA | T | 1 | 22432738 | 22432738 | 5.464 | **0.05** | 22.4 |
|  |  | P | 1 | 44706879 | 44706879 | 10.888 | **0.01** | 44.5 |
|  |  | T:P | 1 | 370379 | 370379 | 0.09 | 0.77 | 0.4 |
|  |  | Residuals | 8 | 32847285 | 4105911 |  |  | 32.7 |
|  | DHA | T | 1 | 1489129 | 1489129 | 9.681 | **0.01** | 41.5 |
|  |  | P | 1 | 826412 | 826412 | 5.373 | **0.05** | 23 |
|  |  | T:P | 1 | 44645 | 44645 | 0.29 | 0.6 | 1.2 |
|  |  | Residuals | 8 | 1230495 | 153812 |  |  | 34.3 |
| *Cyclotella* | EPA | T | 1 | 28817513 | 28817513 | 4.403 | 0.07 | 19.1 |
|  |  | P | 1 | 18242687 | 18242687 | 2.787 | 0.13 | 12.1 |
|  |  | T:P | 1 | 51128167 | 51128167 | 7.811 | **0.02** | 34 |
|  |  | Residuals | 8 | 52365503 | 6545688 |  |  | 34.8 |
|  | DHA | T | 1 | 163 | 163 | 0.007 | 0.94 | 0 |
|  |  | P | 1 | 356382 | 356382 | 14.595 | **0.01** | 61.3 |
|  |  | T:P | 1 | 29952 | 29952 | 1.227 | 0.3 | 5.1 |
|  |  | Residuals | 8 | 195349 | 24419 |  |  | 33.6 |
| *Uroglena* | EPA | T | 1 | 109203 | 109203 | 124.814 | **<0.01** | 86.9 |
|  |  | P | 1 | 7719 | 7719 | 8.823 | **0.02** | 6.1 |
|  |  | T:P | 1 | 1757 | 1757 | 2.009 | 0.19 | 1.4 |
|  |  | Residuals | 8 | 6999 | 875 |  |  | 5.6 |
|  | DHA | T | 1 | 1878457 | 1878457 | 39.637 | **<0.01** | 81 |
|  |  | P | 1 | 1656 | 1656 | 0.035 | 0.86 | 0.1 |
|  |  | T:P | 1 | 59821 | 59821 | 1.262 | 0.29 | 2.6 |
|  |  | Residuals | 8 | 379130 | 47391 |  |  | 16.3 |
| *Synura* | DHA | T | 1 | 43307 | 43307 | 63.38 | **<0.01** | 94.1 |
|  |  | Residuals | 4 | 2733 | 683 |  |  | 5.9 |

**Table S6 EPA and DHA daily gain (µg FA L^-1^ d^-1^) ANOVA results.** Factors: T= temperature, P= phosphorus, T:P= interaction between temperature and phosphorus. Df= degrees of freedom, Sq= sum of squares. In bold p-values <0.05.

| **ANOVA** | **Fatty acid** | **Factor** | **Df** | **Sum Sq** | **Mean Sq** | **F value** | **p-value** | **Explained variance** |
| --- | --- | --- | --- | --- | --- | --- | --- | --- |
| *P. Cinctum* | EPA | T | 1 | 2158 | 2158 | 4.807 | 0.06 | 19.6 |
|  |  | P | 1 | 998 | 998 | 2.222 | 0.17 | 9.1 |
|  |  | T:P | 1 | 4271 | 4271 | 9.514 | **0.02** | 38.8 |
|  |  | Residuals | 8 | 3591 | 449 |  |  | 32.6 |
|  | DHA | T | 1 | 37733 | 37733 | 9.06 | **0.02** | 35 |
|  |  | P | 1 | 2389 | 2389 | 0.574 | 0.47 | 2.2 |
|  |  | T:P | 1 | 34285 | 34285 | 8.232 | **0.02** | 31.8 |
|  |  | Residuals | 8 | 33320 | 4165 |  |  | 30.9 |
| *Rhodomonas* | EPA | T | 1 | 29.9 | 29.9 | 0.768 | 0.41 | 2.3 |
|  |  | P | 1 | 922.5 | 922.5 | 23.651 | **<0.01** | 72.5 |
|  |  | T:P | 1 | 8.7 | 8.7 | 0.224 | 0.65 | 0.7 |
|  |  | Residuals | 8 | 312.1 | 39 |  |  | 24.5 |
|  | DHA | T | 1 | 7.71 | 7.71 | 2.027 | 0.19 | 4.3 |
|  |  | P | 1 | 140.92 | 140.92 | 37.026 | **<0.01** | 77.9 |
|  |  | T:P | 1 | 1.74 | 1.74 | 0.456 | 0.52 | 1 |
|  |  | Residuals | 8 | 30.45 | 3.81 |  |  | 16.8 |
| *Cyclotella* | EPA | T | 1 | 1310 | 1310 | 0.895 | 0.37 | 2.4 |
|  |  | P | 1 | 29177 | 29177 | 19.927 | **<0.01** | 52.4 |
|  |  | T:P | 1 | 13482 | 13482 | 9.208 | **0.02** | 24.2 |
|  |  | Residuals | 8 | 11714 | 1464 |  |  | 21 |
|  | DHA | T | 1 | 0.9 | 0.9 | 0.096 | 0.77 | 0.4 |
|  |  | P | 1 | 121.42 | 121.42 | 12.957 | **0.01** | 52.2 |
|  |  | T:P | 1 | 35.26 | 35.26 | 3.763 | 0.09 | 15.2 |
|  |  | Residuals | 8 | 74.97 | 9.37 |  |  | 32.2 |
| *Melosira* | EPA | T | 1 | 11.3 | 11.3 | 0.601 | 0.46 | 1.5 |
|  |  | P | 1 | 581.6 | 581.6 | 30.941 | **<0.01** | 76.4 |
|  |  | T:P | 1 | 17.6 | 17.6 | 0.938 | 0.36 | 2.3 |
|  |  | Residuals | 8 | 150.4 | 18.8 |  |  | 19.8 |
|  | DHA | T | 1 | 0.146 | 0.146 | 0.468 | 0.51 | 1.1 |
|  |  | P | 1 | 10.293 | 10.293 | 33 | **<0.01** | 74.1 |
|  |  | T:P | 1 | 0.951 | 0.951 | 3.05 | 0.12 | 6.8 |
|  |  | Residuals | 8 | 2.495 | 0.312 |  |  | 18 |

**Table S7 Polyunsaturated fatty acid (PUFA) proportion and daily gain (µg FA L^-1^ d^-1^) Kruskal-Wallis results of groups that presented unequal variances.** Df= degrees of freedom, Sq= sum of squares. In bold p-values <0.05.

| **Kruskal-Wallis** | **Testing** | **Fatty acid** | **Df** | **Chi-squared** | **p-value** |
| --- | --- | --- | --- | --- | --- |
| *Synura* | PUFA proportion |  | 1 | 3.8571 | **0.05** |
| Chlamidomonas | PUFA proportion |  | 3 | 6.3718 | 0.09 |
| *Synura* | Daily gain | DHA | 1 | 2.3333 | 0.13 |
| *Uroglena* | Daily gain | EPA | 3 | 5.8205 | 0.12 |
| *Uroglena* | Daily gain | DHA | 3 | 8.4359 | **0.04** |
